# Blood pressure reduction and RAAS inhibition in diabetic kidney disease: therapeutic potentials and limitations

**DOI:** 10.1007/s40620-020-00803-3

**Published:** 2020-07-17

**Authors:** Giovanna Leoncini, Francesca Viazzi, Salvatore De Cosmo, Giuseppina Russo, Paola Fioretto, Roberto Pontremoli

**Affiliations:** 1 University of Genoa and IRCCS Ospedale Policlinico San Martino, Viale Benedetto 15, 6, 16132 Genoa, Italy; 2 Department of Medical Science, Scientific Institute ‘Casa Sollievo della Sofferenza’, San Giovanni Rotondo, Foggia Italy; 3 Department of Clinical and Experimental Medicine, University of Messina, Messina, Italy; 4 Department of Medicine, University of Padua, Padua, Italy

**Keywords:** Albuminuria, Blood pressure, Diabetic kidney disease, RAAS inhibition, Renal progression

## Abstract

Diabetic kidney disease (DKD) affects approximately one-third of patients with diabetes and taking into consideration the high cardiovascular risk burden associated to this condition a multifactorial therapeutic approach is traditionally recommended, in which glucose and blood pressure control play a central role. The inhibition of renin–angiotensin–aldosterone RAAS system represent traditionally the cornerstone of DKD. Clinical outcome trials have demonstrated clinical significant benefit in slowing nephropathy progression mainly in the presence of albuminuria. Thus, international guidelines mandate their use in such patients. Given the central role of RAAS activity in the pathogenesis and progression of renal and cardiovascular damage, a more profound inhibition of the system by the use of multiple agents has been proposed in the past, especially in the presence of proteinuria, however clinical trials have failed to confirm the usefulness of this therapeutic approach. Furthermore, whether strict blood pressure control and pharmacologic RAAS inhibition entails a favorable renal outcome in non-albuminuric patients is at present unclear. This aspect is becoming an important issue in the management of DKD since nonalbuminuric DKD is currently the prevailing presenting phenotype. For these reasons it would be advisable that blood pressure management should be tailored in each subject on the basis of the renal phenotype as well as related comorbidities. This article reviews the current literature and discusses potentials and limitation of targeting the RAAS in order to provide the greatest renal protection in DKD.

## Introduction

Throughout their lifetime, approximately one-third of patients with diabetes will eventually develop diabetic kidney disease (DKD) [1]. Furthermore, DKD is currently the leading cause of chronic kidney disease as well as of end-stage kidney disease (ESKD) worldwide [2]. In patients with type 2 diabetes, DKD entails an unfavorable impact on cardiovascular (CV) risk burden [3, 4]. As a matter of fact, subjects with diabetes and renal impairment are more likely to die from CV disease than to survive long enough and face the need of renal replacement therapy [5].

In the context of this rather complex and clinically challenging scenario a multifactorial therapeutic approach has traditionally been recommended. As a matter of fact, more than a decade ago, in the Steno-2 study an intensified multifactorial intervention with tight glucose control, the use of renin–angiotensin-aldosterone system (RAAS) blockers, aspirin, and lipid-lowering agents on top of behavior modification had been proved to effectively reduce CV morbidity and mortality over a long-term follow-up in patients with persistent microalbuminuria [6]. Nowadays, a combination of optimal glycemic and blood pressure control by the use of RAAS system inhibitors is still the cornerstone for prevention and treatment of DKD [7].

Hypertension is a common condition in patients with diabetes and its prevalence in subjects with DKD is significantly higher than in general population and increases gradually according to glomerular filtration rate (GFR) decline, reaching the 90% in ESKD subjects [8]. Therefore, the management of hypertension plays a central role since an adequate blood pressure control is able to reduce the burden of albuminuria, the progression of DKD as well as CV risk burden. However, in this setting of intervention, blood pressure targets and pharmacological choices are still a matter of debate in clinical practice.

## Antihypertensive treatment and renal protection in type 2 diabetes: the need for a patient centered approach

**Fig. 1:**
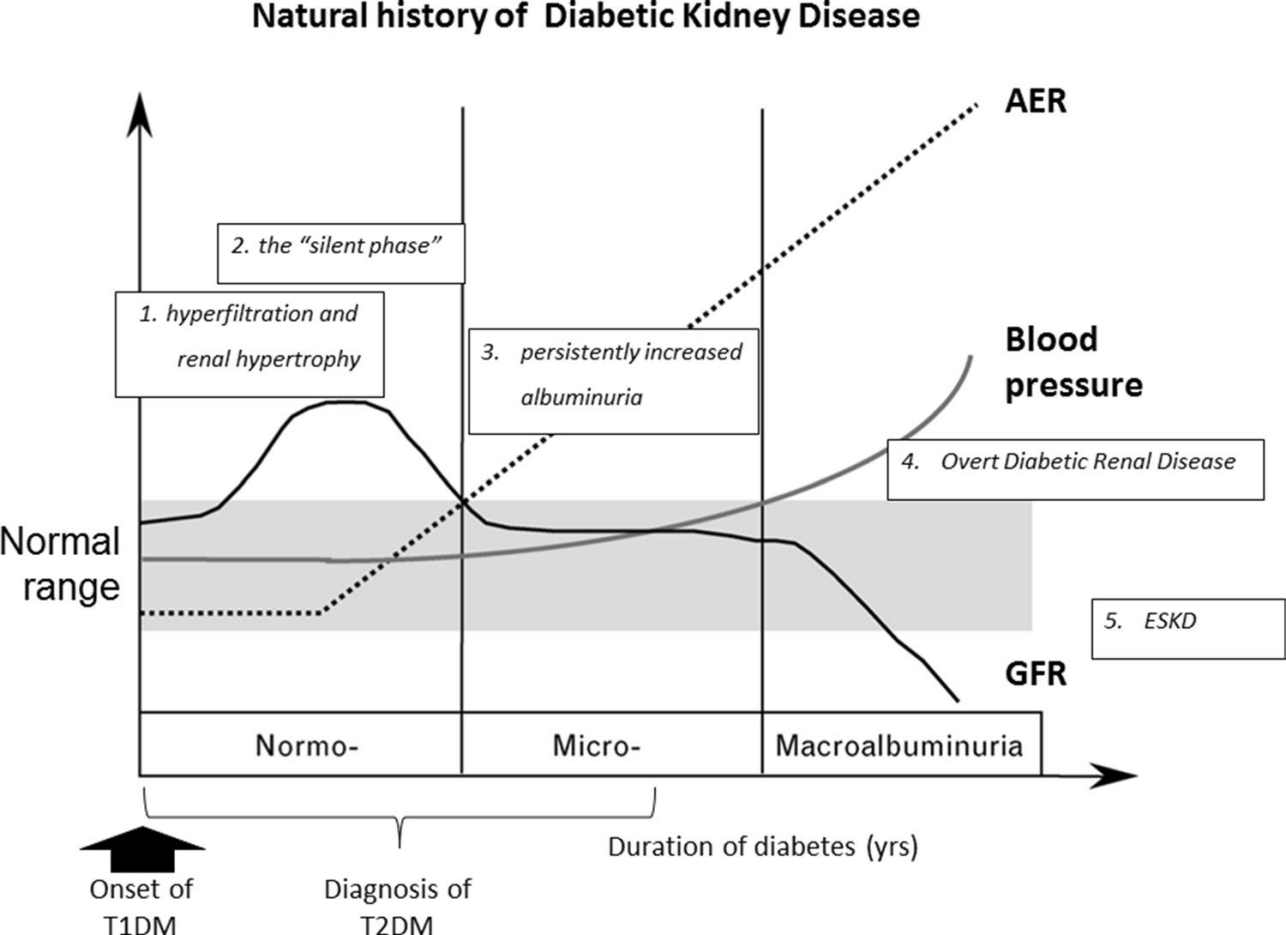
Natural history of diabetic kidney disease

Albuminuria is a known marker of renal damage and has been shown to have a strong cardiovascular and renal predictive power. Furthermore, modifications of urinary albumin excretion under pharmacological treatment might impact CV and renal prognosis independent of blood pressure reduction [9–11]. In the classical, five-stage course of diabetic nephropathy, mostly derived from studies conducted in patients with type 1 diabetes, microalbuminuria has traditionally been taken to represent the first sign of renal involvement [12] (Fig. 1). According to this clinical paradigm, overtime, albuminuria eventually progresses to overt proteinuria, which in turn, precedes GFR decline. While the natural history of DKD in type 2 diabetes seems to be more heterogenous, the assessment of albuminuria has been traditionally considered the reference tool for diagnosis of diabetic nephropathy and the reduction of progression or regression of albuminuria has been the primary end-point in many clinical trials aiming at the evaluation of nephroprotection conferred by the pharmacological inhibition of RAAS [13].

More recent studies, however, have shown that DKD in both type 1 and type 2 diabetes has an heterogeneous histologic phenotype, with various involvement of glomeruli and the interstitium [14]. On the other hand, it has recently been reported that a considerable proportion of diabetic patients with reduced GFR have a normal or only slightly increased urinary albumin excretion indicating that GFR decline does not necessarily parallel changes in albuminuria [15, 16]. Both features of DKD, reduction of GFR and albuminuria, have an independent prognostic role in terms of CV and renal events and, therefore, the awareness and a better knowledge of these different conditions might have an impact in the management of DKD. Several studies as well as real-life observations have pointed out that a strict blood pressure control is associated to a reduction of albuminuria, but is not always associated to a benefit in terms of GFR preservation [17, 18]. Accordingly, it has been suggested that lower BP values might be beneficial in terms of renal protection in diabetic patients with an albuminuric phenotype, while in absence of albuminuria this benefit is uncertain [19]. Therefore, it seems that an individually tailored treatment strategy in terms of both optimal blood targets and choice of antihypertensive drugs combinations could be desirable on the basis of the presenting renal phenotype [20]. This issue needs to be verified by studies specifically conducted in patients with non-albuminuric renal impairment.

DCRecent International Guidelines [21] suggest that in patients with diabetes and hypertension, blood pressure targets should be individualized through a shared decision-making process that addresses cardiovascular risk among other variables. For individuals at high or very high cardiovascular risk (with atherosclerotic cardiovascular disease or 10-year Atherosclerotic Cardiovascular Disease risk ≥ 15%) a blood pressure target of < 130/80 mmHg seems to be appropriate, if it can be safely attained, while for those at lower risk for cardiovascular disease (10-year Atherosclerotic Cardiovascular Disease risk < 15%) a target of < 140/90 mmHg is recommended.

## The inhibition of RAAS along the renal continuum

DKD is a complex disease, wherein various pathological changes including glomerular hypertrophy with progressive mesangial expansion and tubulointerstitial fibrosis are associated with multiple pathophysiological abnormalities such as inappropriate activation of the RAAS at the systemic and tissue level [22, 23]. Hypertension and hyperglycaemia play a central role in the development and progression of renal damage contributing to structural and functional alterations. In particular, at the renal level increase intraglomerular pressure, cellular growth, and fibroblast differentiation have been shown to favor the development of interstitial fibrosis and glomerular sclerosis. Reducing intraglomerular pressure with an angiotensin converting enzyme inhibitor (ACEI) or angiotensin receptor blocker (ARB) can minimize, or even prevent, glomerular disease and albumin excretion. Furthermore, several experimental, animal, and in vivo studies have shown that the pharmacologic inhibition of RAAS leads to attenuation of interstitial fibrosis and glomerular sclerosis by down-regulation of advanced glycation end-products, TGF-β, NADPH oxidase, reactive oxygen species, reduced expression of receptors for advanced glycation end-products, reduced type IV collagen excretion and reduced mesangial extracellular matrix accumulation [24]. For all these reasons RAAS inhibitors have traditionally represented the mainstay treatment of hypertension in diabetic patients [25]. In fact, the effect of pharmacological RAAS blockade at various levels, by using ACEIs, ARBs, direct renin inhibitors, and mineralocorticoid antagonists, has been evaluated along the renal continuum.

### Potentials of RAAS Inhibition

Main clinical trials that have evaluated renal protective effect of RAAS blockade across the renal continuum in DKD are reported in Table 1.

**Table 1:** Major clinical trials on pharmacologic RAAS inhibition and nephroprotection in DKD

References	Population	Comparison groups	Follow-up	Results	Comments	Conclusion
*Prevention of increased albuminuria (single RAAS inhibitor)*						
Ravid et al. (1998) [60]	156 normotensive and normoalbuminuric patients with T2DM	Enalapril vs placebo	6 years	Enalapril treatment resulted in an absolute risk reduction of 12.5% (95% CI, 2–23%; P = 0.042) for development of microalbuminuria	Mean blood pressure remained normal (< 107 mm Hg) in all patients. Mean decrease in creatinine clearance 0.025 mL/s per year in enalapril group and 0.04 mL/s per year in placebo group (P = 0.040)	ACEI attenuated the decline in renal function and reduced the extent of albuminuria in normotensive, normoalbuminuric patients with T2DM
BENEDICT (Bergamo Nephrologic Diabetes Complication Trial) (2004) [29]	1204 normoalbuminuric T2DM patients	Trandolapril (2 mg/day) + Verapamil (sustained-release formulation, 180 mg/die) *vs* Trandolapril alone (2 mg/day) *vs* Verapamil alone (sustained-release formulation, 240 mg/day) *vs* placebo	3.6 years	Trandolapril delayed the onset of microalbuminuria (HR 0.47 95% CI 0.26–0.83)	The effect on the onset of microalbuminuria was significant even after adjustment for baseline and follow-up systolic and diastolic blood pressures	In normoalbuminuric patients with T2DM and hypertension the use of ACEI plus verapamil and ACEI alone decreased the incidence of microalbuminuria to a similar extent
ADVANCE (The Action in Diabetes and Vascular Disease: Preterax and Diamicron-MR Controlled Evaluation) (2007) [61, 62]	11,140 patients with T2DM and either normoalbuminura or microalbuminuria	ixed combination of Perindopril-Indapamide vs placebo	4.3 years	Active treatment reduced the risk for progression of albuminuria among patients who had either normo- or microalbuminuria at baseline (HR 0.78; 95% CI 0.72–0.84; P < 0.0001) and the risk for new-onset microalbuminuria in patients with normoalbuminuria at baseline (HR 0.79; 95% CI 0.73–0.86; P < 0.0001)	Active treatment reduced the risk to develop the composite renal outcome of new-onset microalbuminuria, new-onset nephropathy, doubling of serum creatinine above 200 μmol/L, or end-stage kidney disease (HR 0.79; 95% confidence interval [CI] 0.73–0.85; P < 0.0001)	The combined treatment ACEI + diuretic reduced the risk for developing a renal outcome among patients with T2DM as well as new onset of microalbuminuria alone
RASS (Renin Angiotensin System Study) (2009) [26]	285 normotensive normoalbuminuric T1DM patients	Losartan (100 mg/day) vs Enalapril (20 mg/day) vs placebo	5 years	The cumulative incidence of microalbuminuria was 6% in the placebo group; the incidence was higher with Losartan (17%, P = 0.01 by the log-rank test) but not with Enalapril (4%, P = 0.96 by the log-rank test).	Treatment with either Losartan or Enalapril had no effect compared to placebo on the fraction of glomerular volume occupied by the mesangium (the primary study end point) or other histologic findings seen in DKD. However, they found benefit on retinopathy progression of both enalapril and losartan as monotherapy over placebo	Early use of ACE-I or ARB in T1DM patients did not slow nephropathy progression but slowed the progression of retinopathy
DIRECT (Diabetic Retinopathy Candesartan Trials) (2009) [27]	3326 T1DM and 1905 T2DM normoalbuminuric patients	Candesartan (16–32 mg/day) vs placebo	4.7 years	No effect of candesartan on risk for microalbuminuria (pooled hazard ratio, 0.95 [95% CI, 0.78–1.16]; P = 0.60). Annual rate of change in albuminuria was 5.53% lower (CI, 0.73–10.14%; P = 0.024) with Candesartan than with placebo	Investigators recruited Mainly normotensive patients or patients with well-controlled hypertension with a low overall vascular risk were recruited and, therefore, a low rate of microalbuminuria was observed	ARB did not prevent microalbuminuria in mainly normotensive patients with T1DM or T2DM
ROADMAP (Randomized Olmesartan and Diabetes Microalbuminuria Prevention) (2011) [30]	4447 T2DM patients	Olmesartan (40 mg/day) *vs* placebo	3.2 years	Olmesartan resulted in a reduction in time to micro-albuminuria onset by 23% (HR 0.77; 95% CI 0.63–0.94; P = 0.01). Olmesartan was also effective in delaying the onset of microalbuminuria (8.2% versus 9.8%) independently of blood pressure	Blood pressure was similarly controlled in both study arms	ARB delayed onset of microalbuminria compared to placebo
*Prevention of severely increased albuminuria*-*overt Diabetic Kidney Disease (single RAAS inhibitor)*						
The Microalbuminuria Captopril Study Group (1996) [63]	235 normotensive patients with T1DM and persistent microalbuminuria	Captopril *vs* placebo	2 years	Progression to overt proteinuria was markedly reduced in the patients treated with Captopril (7.6% versus 23.1%)	This degree of risk reduction remained similar after adjustment for differences in time-varying mean arterial blood pressure	ACEI reduces the risk of progression to overt nephropathy in IDDM patients with microalbuminuria
HOPE and MICRO-HOPE (2000) [28]	3577 with diabetes at increased risk of cardiovascular events without clinical proteinuria	Ramipril *vs* placebo	5 years	Ramipril reduced the risk of overt nephropathy by 24% (95% CI 3–40, p = 0.027)		ACE reduced the risk of overt nephropathy in diabetic patients
IRMA-2 (The Irbesartan in Patients with Type 2 Diabetes and Microalbuminuria Trial) (2001) [31]	590 hypertensive, microalbuminuric T2DM patients	Irbesartan (150–300 mg/day)	2 years	Treatment with Irbesartan (150–300 mg/day) was associated with a dose-dependent reduction in risk of progression to macroalbuminuria, with an almost threefold risk reduction with the highest dose (300 mg/day) at 2 years of follow-up (HR 0.30 95% CI 0.14–0.61)	This effect was independent of the blood pressure-lowering properties of irbesartan. A lower irbesartan dose of 150 mg per day had no appreciable effect on macroalbuminuria incidence	ARB reduced the risk of overt nephropathy independently of its blood-pressure-lowering effect in patients with T2DM and microalbuminuria
MARVAL Trial (2003) [64]	332 patients with T2DM and microalbuminuria, with or without hypertension	Valsartan (80 mg) *vs* Amlodipine(5 mg)	24 weeks	The primary end point was the percent change in UAER from baseline to 24 weeks. Albuminuria at 24 weeks was 56% (95% CI, 49.6–63.0) of baseline with valsartan and 92% (95% CI, 81.7–103.7) of baseline with amlodipine, with highly significant between-group effect (P < 0.001). Valsartan lowered UAER similarly in both the hypertensive and normotensive subgroups. More patients reversed to normoalbuminuria with valsartan (29.9% versus 14.5%; P = 0.001)	Over the study period, BP reductions were similar between the two treatments	ARB reduced significantly albuminuria respect to amlodipine
DETAIL trial (2004) [65]	250 patients with T2DM and early nephropathy (82% moderately increased albuminuria and 18% severely increased albuminuria to a maximum of 1.4 g/d) and a baseline GFR GFR > 70 mL/min/1.73 m^2^	Enalapril 20 mg *vs* Telmisartan 80 mg	5 years	The mean change in GFR with Telmisartan and Enalapril was −17.5 mL/min per 1.73 m^2^ and −15.0 mL/min per 1.73 m^2^, respectively, within the statistical bounds of noninferiority Telmisartan and Enalapril similarly decreased blood pressure and albuminuria	There was a high dropout rate (approximately one-third)as a consequence of adverse events, however the number of dropouts and reasons for withdrawal were similar in the two treatment groups	ARB and ACEI provided similar renal protection
INNOVATION (Incipient to Overt: Angiotensin II Blocker, Telmisartan, Investigation on Type 2 Diabetic Nephropathy) (2007) [66]	514 T2DM patients aged from 30 to 74 years with urinary albumin-to-creatinine ratio 100–300 mg/g and serum creatinine < 1.5 mg/dayl (men) and < 1.3 mg/dl (women).	Telmisartan (80 mg or 40 mg) vs placebo	1.3 years	Telmisartan was associated with a lower transition rate to overt nephropathy (transition rates with Telmisartan 80 mg 11.0%, Telmisartan 40 mg telmisartan 21.0%, and placebo 44.2%; both telmisartan doses *vs* placebo, P < 0.01)	After adjustment for the difference in blood pressure levels between the placebo and treatment groups, the beneficial effect of telmisartan in delaying progression to overt nephropathy persisted	ARB reduced transition rates to overt nephropathy, compared with placebo
*Prevention of progression to ESKD (single RAAS inhibitor)*						
Collaborative Study (1993) [32]	409 T1DM patients with DKD proteinuria ≥ 500 mg/die and creatinine ≤ 2.5 mg/dl	Captopril (25 mg three times/d) *vs* placebo	3 years	Captopril was associated with a 48% reduction of doubling creatinine (HR 0.52 95% CI 0.36–0.84, P = 0.007)	Multivariate analyses showed that the treatment effect was independent of the small blood pressure differences between the two groups and could be explained by the reduction in urinary protein excretion associated with captopril treatment. No significant benefit was observed in patients with baseline serum creatinine < 1.5 mg/dL	ACE-I protected against the progression of DKD in T1DM
RENAAL (The Reduction of Endpoints in NIDDM with the Angiotensin II Antagonist Losartan Study) trial (2001) [33]	1513 T2DM patients with overt nephropathy (urine protein/creatinine ≥ 300 mg/g and creatinine level between 1.3 and 3 mg/dL)	Losartan (50–100 mg/day) *vs* placebo	3.4 years	Losartan was associated with a 16% reduction of a composite end point of doubling of serum creatinine concentration, ESRD, or death (HR 0.84 95% CI 0.72–0.98). In particular, the doubling of plasma creatinine by 25% and ESRD by 28%, and Losartan reduced albuminuria by 28%, while the placebo was associated with a 4% increase in albuminuria, in the first 6 months	The effect remained significant even after adjustment for the small differences in blood-pressure control between treatment groups	ARB protect against the progression of DKD in T2DM
IDNT (Irbesartan Diabetic Nephropathy Trial) (2001, 2005) [34, 67]	1715 hypertensive T2DM patients with overt nephropathy (proteinuria ≥ 900 mg and a creatinine between 1 and 3 mg/dL)	Irbesartan (300 mg/day) *vs* amlodipine (10 mg/day) *vs* placebo	2.6 years	Irbesartan was associated with a 23% reduction of the primary endpoint (doubling of the plasma creatinine, development of ERSD, or death from any cause) respect to Amlodipine (HR 0.77 95% CI 0.63–0.93, P = 0.005) and 19% reduction respect to placebo (HR 0.81 95% CI 0.67–0.99, P = 0.03). A post hoc analysis reported significantly lower levels of proteinuria were obtained with Irbesartan (41% average decrease) than with Amlodipine (11%) or control (16%) at 1 year	Results remained significant even after adjustment for the small differences in blood-pressure control between treatment groups	ARB is effective in protecting against the progression of DKD in T2DM
ORIENT (Olmesartan Reducing Incidence of End-stage Renal Disease in Diabetic Nephropathy Trial) trial (2011) [68]	577 Japanese and Chinese patients with T2DM and overt nephropathy	Olmesartan *vs* placebo	3.2 years	Olmesartan did not reduce the risk of the composite renal outcome (doubling of serum creatinine concentration, ESRD or death) (HR 0.97, 95% CI 0.75–1.24; p = 0.791)	77% of the ORIENT study population was receiving ACEI therapy at baseline and this therapy was continued throughout the trial period	ARB did not improve progression of DKD in T2DM on top of ACEI
*Dual RAAS Blockade with ACEI/ARB*						
CALM (Candesartan and Lisinopril Microalbuminuria) (2000) [69]	199 hypertensive, T2DM and microalbuminuric patients	Lisinopril (20 mg) *vs* Candesartan (16 mg) *vs* their combination	12 weeks	Combination therapy more effective with greater reduction in urinary albumin: creatinine ratio (50%, 36–61%, P < 0.001) compared with candesartan (24%, 0–43%, P = 0.05) or lisinopril (39%, 20–54%, P < 0.001) alone	Dual therapy resulted also in a greater reduction in systolic and diastolic BPs, over monotherapy (P < 0.001)	Equivalent reduction of BP and microalbuminuria between ACE and ARB monotherapy. Combination therapy further decreased microalbuminuria with reduction of BP
Jakobsen et al. (2002) [70]	24 patients with T1DM and DKD	Placebo + enalapril 40 mg/day vs irbesartan 300 mg/day + enalapril 40 mg/day	8 weeks	Further 25% reduction in albuminuria when adding irbesartan (300 mg/day) on top on enalapril respect to placebo/(P < 0.001)	Dual therapy resulted also in a greater reduction in systolic and diastolic BPs, over placebo + enalapril (P < 0.01)	Combination therapy (ACEI + ARB) is superior to maximal recommended dose of ACEI in reducing albuminuria and blood pressure in T!DM patients
IMPROVE (The Irbesartan in the Management of Proteinuric Patients at High Risk for Vascular Events) (2007) [71]	405 patients with T2DM (89%), hypertension, and albuminuria	Combination of Ramipril (10 mg/day) + Irbesartan (300 mg/day) *vs* Ramipril (10 mg/die) + placebo	20 weeks	Adjusted week 20 baseline geometric ratios for Ramipril + Irbesartan and Ramipril + placebo were not significantly different	Although relatively small, the reduction in both diastolic and systolic blood pressure was slightly, but significantly greater in patients receiving ramipril + irbesartan compared with those receiving ramipril + placebo. However these changes in BP did not affect albuminuria	Dual RAAS blockade is not more nephroriotective than monotherapy in patients with cardiovascular risk and microalbuminuria
ONTARGET (Ongoing Telmisartan Alone and in combination with Ramipril Global Endpoint) (2008) [39, 72]	25,620 patients > 55 years with established cardiovascular disease (including 9603 patients with T2DM)	Ramipril (10 mg/day) + Telmisartan (80 mg/day) *vs* Ramipril alone	56 months	Increase in urinary albumin excretion was less with Telmisartan (p = 0·004) or with combination therapy (p = 0·001) than with Ramipril	In 3163 patients with DKD, combination therapy was associated with a similar death rate (2.3% versus 2.2%) and higher rates of acute kidney injury requiring dialysis (1.4% versus 0.8%), hyperkalemia (11.3% versus 7.8%), hypotension (2.8% versus 1.9%) and diarrhea	Although combination therapy of ACEI + ARB reduces proteinuria to a greater extent than monotherapy is associated with more adverse events without and overall worens major renal outcomes
VA NEPHRON-D (Veterans Affairs Nephropathy in Diabetes) (2013) [38]	1448 mostly male T2DM patients with DKD (macroalbuminuria and moderate-to-severe renal impairment (eGFR 30–90 mL/min/1.73 m^2^)	Losartan (100 mg/day) + lisinopril (10–40 mg/day) *vs* losartan alone	Terminated at 2.2 years	The occurrence of primary end point (first occurrence of a decline eGFR of ≥ 30 mL/min/1.73 m^2^ if baseline eGFR ≥ 60 mL/min/1.73 m^2^ otherwise a decline of ≥ 50%, ESKD or death) was similar (HR with combination therapy, 0.88, 95% CI 0.70–1.12; P = 0.30)	A significantly higher frequency of acute kidney injury requiring hospitalization (18% versus 11%) or severe hyperkalemia (9.9% versus 4.4%) without renal benefit	Combination therapy with an ACE inhibitor + ARB was associated with an increased risk of adverse events among patients with DKD
*Dual RAAS blockade with ACEI/ARB and direct renin inhibitor (DRI)*						
AVOID (Aliskiren in the Evaluation of Proteinuria in Diabetes) (2008) [73]	599 T2DM patients with hypertension and nephropathy (macroalbuminuria)	Aliskiren (300 mg/day) vs aliskiren plus losartan (100 mg/day)	24 weeks	Combination therapy was associated with a significant 20% (95% CI 9–30; P < 0.001) greater reduction in albuminin to creatinine ratio. No significant difference in the rate of decline in eGFR were reported	Aliskiren therapy was associated with a significant increase in the risk of hyperkalemia A small, not significant difference in blood pressure was seen between the treatment groups by the end of the study period with lower values in Aliskiren group	DRI may have renoprotective effects that are independent of its blood-pressure-lowering effect in T2DM patients with DKD receiving the recommended renoprotective treatment
ALTITUDE (Aliskiren Trial in Type 2 Diabetes Using Cardiorenal Endpoints) (2012) [40]	8561 T2DM patients	All patients receiving an ACEIs or ARBs at baseline, randomly assigned to aliskiren (300 mg/day) *vs* placebo	Terminated at 2.7 years	Incidence of kidney or cardiovascular events was similar with aliskiren and placebo (6% versus 5.9%)	Adverse events requiring cessation of therapy (worsening of renal function, hyperkalemia, hypotension and diarrhea) were significantly more frequent (13.2% versus 10.2%) in the Aliskiren group	The addition of DRI to standard therapy with RAAS blockade in patients with T2DM who are at high risk for cardiovascular and renal events is not beneficial and may even be harmful

*Patients with normal albuminuria* In diabetic patients without microalbuminuria RAAS blockade has been studied to evaluate the effect on the transition from normoalbuminuria to microalbuminuria. Trials conducted on type 1 diabetic patients, such as Renin-Angiotensin System Study (RASS) [26] and Diabetic Retinopathy Candesartan Trial (DIRECT)-Prevent 1, and DIRECT-Protect 1 [27] failed to show any benefit in the prevention of the development of microalbuminuria. In patients with type 2 diabetes results are inconclusive. In the HOPE (Heart Outcomes Prevention Evaluation) trial ramipril was not effective [28], while in the the Bergamo Nephrologic Diabetes Complications Trial (BENEDICT) [29] the 2 arms containing trandolapril showed similar benefit in preventing the development of albuminuria, and their effect seemed to be independent of blood pressure reduction. Furthermore, in the Randomized Olmesartan and Diabetes Microalbuminuria Prevention (ROADMAP) [30] the use of olmesartan prevented or delayed the onset of microalbuminuria, but difference in blood pressure between the olmesartan and placebo arms were statistically different.

*Patients with moderately increased albuminuria* As for the transition from microalbuminuria to overt proteinuria in the IRMA-2 (Effect of Irbesartan in the Development of Diabetic Nephropathy in Patients With T2DM) trial [31] irbesartan reduced the risk for the development of overt proteinuria (defined as albumin excretion > 200 mg/day) and the effect was dose-dependent.

*Patients with severely increased albuminuria* More solid data support the use of RAAS inhibitors in DKD with overt albuminuria. The Collaborative study group evaluated the impact of captopril versus placebo on CKD progression in patients with type 1 diabetes and proteinuria [32]. Despite similar blood pressure control the captopril group had a significantly lower rates of chronic kidney disease progression or ESKD. Similarly, the data from the RENAAL (Reduction of Endpoints in NIDDM with the Angiotensin II Antagonist Losartan) [33] and IDNT (Irbesartan Diabetic Nephropathy Trial) [34] trials confirmed the nephroprotective effect of ARBs, in patients with DKD with proteinuria.

Therefore, the use of RAAS blockers has been considered for many years the gold standard therapy to reduce cardiovascular risk and to slow the progression of diabetic nephropathy and previous guidelines recommended the use of RAAS blockers in all patients with diabetes without distinction [35]. However, while lower BP values and pharmacologic inhibition of the RAAS have consistently been shown to improve renal outcome in diabetic patients presenting with the traditional “albuminuric” renal phenotype, it is uncertain whether a similar therapeutic strategy should be applied in the absence of albuminuria. In fact, RCTs as well as real-life reports have demonstrated that the use of RAAS inhibiting drugs in patients with diabetic nephropathy has a clinical significant benefit in slowing renal progression only in people with increased albuminuria.

Accordingly, more recently, guidelines do not recommend routine use of RAAS blockers in diabetic subjects unless in the presence of nephropathy with an urinary albumin-to-creatinine ratio greater than 30 mg/g creatinine in order to reduce the risk of progressive kidney disease [21, 36, 37]. Either an ACEI or an ARB are recommended, as these drug classes have shown similar nephroprotective effect. ARB have been studied more extensively than ACEi in patients with type 2 diabetes; on the other hand, no study has been completed with ARB in type 1 diabetes. As for dosage, in the absence of side effects the maximum tolerated dose approved for blood pressure treatment is usually recommended. While in the absence of albuminuria a less strict blood pressure control could be advisable and RAAS inhibitors may not necessarily represents the preferred antihypertensive drugs and treatment for hypertension should include drug classes demonstrated to reduce cardiovascular events in patients with diabetes (ACE inhibitors, angiotensin receptor blockers, thiazide-like diuretics, or dihydropyridine calcium channel blockers) without cogent preference [21, 25].

### Limitations of RAAS inhibition

Several studies have explored the potential benefit of maximal inhibition of the RAAS by means of high doses monotherapy or combination drug. Dual RAAS blockade is based on the pathophysiological rationale of a more profound inhibition of the RAAS cascade which may counteract the well known “aldosterone escape” phenomenon observed in patients treated with a single agent. However, disappointing results from several mega trials with hard endpoints have subsided the initial enthusiasm on the promising effects of dual RAAS blockade on surrogate outcomes. In fact, the combination of two RAAS blocking drugs did not provide considerable benefit in terms of long term renal protection and was accompanied by higher incidence of acute kidney injury and hyperkaliemia as compared to single-drug therapy [38–40]. It is likely that dual/multilevel RAAS blockade completely abolishes the protective role of this system in maintaining renal haemodynamics under stressful conditions and, therefore, may favor AKI in hypovolemic patients. Accordingly, international guidelines currently discourage the use of dual RAAS blockade for the prevention and treatment of DKD [21, 25].

Finally, mineralocorticoid receptor inhibitors (MRI) such as spironolactone or eplerenone might offer an additional opportunity for RAAS intervention by counteracting the “aldosterone breakthrough” that occurs in a subset of patients on RAAS-inhibiting therapy. Aldosterone is known to affect volume status by the regulating renal sodium reabsorption and exert profibrotic effects through increased production of TGF-beta, reactive oxygen, species, PAI-1 and increased collagen gene expression and synthesis [41, 42] and, the use of MRI has been demonstrated to reduce inflammation and fibrosis at kidney level [43–45]. Several studies have shown that the addition of aldosterone receptor blockade to ACEI or ARB blockade can lead to further reduction in albuminuria including in DKD [46–48], however long-term data on the efficacy of mineralocorticoid receptor antagonists on hard endpoints, for example the development of ESKD or patient survival, are still lacking.

## Conclusions

Multiple trials have demonstrated that the use of RAAS blocking drugs can slow progression to ESKD in patients with diabetic nephropathy and proteinuria > 300 mg/day, however single agent RAAS blockade is not sufficient in slowering progression in many patients. In fact, significant residual risk remains in patients with DN and many patients progress to ESKD despite standard recommended care, including optimal control of blood pressure, glycemia and lipids and the use of RAAS blockade [49, 50]. Furthermore, a paradoxical J-curve relationship between BP reduction and renal morbidity may limit the benefit of aggressive treatment strategies, especially in elderly patients with a renal dysfunction without albuminuria [51]. Thus, different individually tailored therapeutic targets, that take into account specific cardiovascular and renal phenotype should guide therapeutic strategies.

Given the steady increase in the prevalence of diabetes worldwide and the soaring costs related to its management, prevention and treatment of DKD remains an unmet clinical need. Within this context, recently developed therapeutic agents such as sodium-glucose co-transporter-2 inhibitors and glucagone like peptide-1 receptor agonist have shown promising therapeutic potential not only to reduce CV risk but also to slow the progression of kidney disease with beneficial effect on albuminuria as well as on GFR [52–55]. As a matter of fact, the nephroprotective effect of these drug classes, especially SGLT2-is, was shown to be additive to RAAS-is and consistent in various studies completed so far, irrespective of baseline GFR and the presence of albuminuria. Several mechanisms, both glycemic and extra glycemic, have been proposed to account for the observed benefit on renal and cardiovascular events [56–59].

Because of their remarkable antiproteinuric and renoprotective activity, confirmed in many large trials, SGLT2i may soon become, in association with RAASi, the standard of care to prevent DKD and retard its progression.

## Take home messages

- Diabetic kidney disease (DKD) is the leading cause of end-stage kidney disease and represent a significant cause of morbidity and mortality worldwide due to the related cardiovascular risk burden.
- The standard of care for management of DKD over the last three decades has been the control of cardiovascular risk factors and the pharmacologic inhibition of renin–angiotensin–aldosterone system (RAAS).
- Nowadays, non-albuminuric DKD is the prevailing phenotype. The nephroprotective role of intensive blood pressure control and the use of RAAS inhibitor in these patients is at present unclear.
- Recent evidence supports a renal protective effect of sodium-glucose cotransporter 2 (SGLT2) inhibitors independent of their hypoglycemic properties.
- We suggest that co-treatment with RAAS and SGLT2 inhibitors may represent the new era of therapeutic options for DKD.
